# Identification of clinically related requirements of a novel assistive device for people with a high spinal cord injury

**DOI:** 10.1371/journal.pone.0218393

**Published:** 2019-06-28

**Authors:** Amihai Gottlieb, Meir Plotnik, Racheli Kizony, Zoe Katsarou, Sevasti Bostantjopoulou, Gabi Zeilig

**Affiliations:** 1 Center of Advanced Technologies in Rehabilitation, Sheba Medical Center, Ramat Gan, Israel; 2 Department of Neurological Rehabilitation, Sheba Medical Center, Ramat Gan, Israel; 3 Department of Physiology and Pharmacology, Sackler Faculty of Medicine, Tel Aviv University, Tel Aviv, Israel; 4 Sagol School of Neuroscience, Tel Aviv University, Tel Aviv, Israel; 5 Department of Occupational Therapy, University of Haifa, Haifa, Israel; 6 Neurology, Hippokration Hospital, Thessaloniki, Greece; 7 3rd Department of Neurology, University of Thessaloniki, Thessaloniki, Greece; 8 Department of Physical Medicine & Rehabilitation, Sackler Faculty of Medicine, Tel Aviv University, Tel Aviv, Israel; University of Rome, ITALY

## Abstract

People with spinal cord injuries (SCI), and particularly with high level lesions, can potentially lose the ability to effectively operate computers. The Multimedia Authoring and Management using your Eyes and Mind (MAMEM) project aims to design and produce a novel assistive device to support computer use by individuals with SCI and other disabilities. The solution harnesses eye tracking and brain waves, as measured by encephalography (EEG), to manipulate common computer functions. This paper describes the first step in the project, during which we defined clinically related requirements of the assistive device. These definitions were based on data from three sources: (1) a narrative review; (2) a focus group of SCI rehabilitation professionals; and (3) structured questionnaires administrated to potential computer users with SCI, addressing computer-use habits, barriers, and needs. We describe both the collection of data from each source and the clinically related requirements extracted. The novel three-source requirement assessment method is discussed, and the advantages and disadvantages of each data source are reported. In conclusion, we suggest that this approach makes it possible to organize, discuss, and prioritize the requirements, and to create a work program while planning the device. This increases our level of certainty that the efficacy and adequacy of the assistive device will be maximized, in terms of the clinical needs of users.

## Introduction

Loss of voluntary upper extremity control alongside preserved cognitive function is common in persons with high spinal cord injuries (SCI), and can result in difficulty operating computers [1, 2]. As computers play an important role in everyday life, and specifically in social participation, the ability to control them can have significant bearing on quality of life (QOL)[3]. In particular, affected individuals may find themselves marginalized and unable to stay maintain social ties.

A recent study conducted in the Netherlands [4] showed that among 265 individuals with SCI, those with tetraplegia used one or more assistive devices to access their computers, mostly typing aids or trackballs. However, the use of speech-recognition software and head or mouth controls was low, probably because few people with C4 SCI were included in the study. In the United States, a study [5, 6] conducted between 2003 and 2006 showed that only 35% of participants with tetraplegia used an assistive device to enable computer and Internet use, despite the ability of these devices to facilitate Internet use [7, 8]. These studies highlight the need for novel interfaces that compensate for loss of body functions by utilizing other available input sources for control [7]. During the past few years, potential input sources and methods for operating computers or assistive devices have been suggested, such as embodiment of tools [9], brain-computer interactions [10], eye-tracking [11], and more.

In line with this idea, the European Union (EU) funded a project entitled *Multimedia Authoring and Management using your Eyes and Mind (MAMEM)* [12] with the overarching goal of facilitating social integration among individuals with disabilities by improving their ability to use computers. To achieve this aim, the project focused on delivering technologies that enable operation of software applications and execution of multimedia tasks using novel, alternative interface channels such as eye movements and EEG signals.

### Requirements of assistive technologies for controlling computers

To date, new assistive technologies for controlling computers (ATCC), such as joysticks for the hand or chin, head-trackers, and even tongue-controlled interfaces have been developed for individuals with disabilities, among them people with SCI [13–15]. However, the rates of abandonment (disuse of a previously obtained device, for any reason) of assistive devices are quite high, reaching up to 60% [16–21]. Several explanations have been proposed for these rates [20], which have a clear detrimental impact [17]. One possible cause is lack of end user involvement in planning and decision-making within the processes of designing or purchasing an ATCC [17, 22]. Indeed, in recent years, the involvement of end users in designing or choosing of an assistive device has risen [1, 7, 20, 23–27] and the methods used in these requirement definition studies include reviews of medical records [20], interviews [24, 25], questionnaires [1, 7, 27], and various qualitative methods [23, 26]. The MAMEM project adopted a similar approach for the definition of ATCC requirements.

### Requirement assessment in the MAMEM project

The MAMEM project targeted three end user groups: people with a high SCI, people with Parkinson's disease, and people with neuromuscular disorders. Here, we describe the process of defining the system requirements specifically addressing the needs of persons with a high SCI. Information was gathered from several sources, to identify as many clinically related personal preferences and as much existing knowledge as possible. Our objective was to produce a final, consolidated set of requirements related to persons with a high SCI. To this end, we administered questionnaires to potential end users, conducted focus group sessions with health professionals, and surveyed up-to-date information that has accumulated in the scientific literature.

## Materials and methods

We collected data from three sources: (1) a literature survey conducted in the format of a narrative review; (2) a focus group with various health professionals in the field of SCI rehabilitation; and (3) structured questionnaires administrated to potential computer users with SCI, addressing computer-use habits, barriers, and needs. Data from these three sources were used to extract clinically related requirements for the platform.

### Literature survey method: Narrative review

To efficiently extract clinically related requirements from the literature, we conducted a narrative review [28], in which we appraised current knowledge presented in review articles and papers explicitly discussing the computer operation challenges and difficulties of people with SCI. Our goal was to identify clinically related requirements as efficiently as possible.

We conducted an electronic search in online databases (PubMed, Google Scholar) by crossing the terms: "Spinal cord injury," "tetraplegia," "computer use habits," "computer use difficulties," "computer use needs," and "assistive devices." To be included in the analysis, articles had to be written in English and published between the years 2000 and 2015. Potential to provide data that could be used to extract clinically related requirements was assessed based on article abstracts. There were no exclusion criteria.

#### Extracting outcomes from narrative review

The final set of articles (see Results section) was critically read and reviewed. Clinically related requirements were derived from the original conclusions and inferred from the text.

### Focus group method

Our focus group method was based on the guidelines of the consolidated criteria for reporting qualitative research (COREQ), a 32-item checklist for interviews and focus groups [29]. Focus group participants comprised a convenience sample of health professionals who work closely with persons with SCI in a rehabilitation hospital. All were approached by email and none refused to participate. The sample included the following six professionals: a physician boarded in physical medicine and rehabilitation; a registered rehabilitation nurse, a social worker, a rehabilitation psychologist, an occupational therapist, a physical therapist, and a speech therapist. All worked together at the same rehabilitation hospital prior to participation and had extensive expertise in the rehabilitation of people with SCI. To guide the group and transcribe the content raised, a commercial company (TNS ltd., Tel-Aviv, Israel) specializing in focus groups was hired. The guide was held a graduate degree and had 14 years of experience in qualitative research, including focus groups. No incentives for attendance were provided. The focus group took place in a single, two-and-a-half-hour session in a quiet seminar room in the rehabilitation hospital. The discussion was taped and video-recorded, with participant consent.

During the session, participants were first informed of the purpose of the focus group in the context of the MAMEM project research plan and timeline. The guide then provided information about herself and her role and explained how participants were expected to behave during the focus group and why certain unproductive behaviors (e.g., refraining from contradicting a superior) should be avoided. Once started, the focus group was led by the guide, who introduced a topic and related questions and asked each participant to provide their input. Two main topics were raised: (1) people with high tetraplegia and their computer habits, including preferred ATCC; and (2) barriers encountered by people with high tetraplegia when dealing with ATCC and computers.

#### Focus group results analysis

The focus group recordings were transcribed and then analyzed by a professional focus group content analyzer. The analysis was conducted in accordance with a standard content analysis method, in which words, sentences, or paragraphs relevant to the main questions were identified. These text units were then further analyzed to identify common themes, which were then condensed into statements reflecting the common ideas raised by the group. Finally, we interpreted these statements to extract clinically related requirements.

### Questionnaire-based cross-sectional study method

#### Participants

Included participants were 18–80 years old, with chronic (i.e., no longer hospitalized due to injury) SCI. Inclusion criteria included presence of complete or incomplete SCI (ASIA score A, B, C, or D) with a neurological level of injury of C5 and above (level of spinal sever was on or above vertebra C5), and tetraplegia [30]. Overall, 23 individuals were recruited from the outpatient clinics at the Sheba Medical Center Rehabilitation Center. Their demographic and clinical information appear in Tables 1 and 2. They were screened over the phone and those who were eligible entered the study. The study protocol was approved by the Sheba Medical Center Institutional Review Board, and all participants signed informed written consent prior to entering the study.

**Table 1:** Demographic data of the participants in the questionnaire study.

	N	Mean (SD) / %
Age	23	49.2 (15.8)
Education (years)	23	14.9 (4.6)
Gender		
Male	16	69%
Marital status		
Married	16	26%
Single	6	70%
Widowed	1	4%
Number of children	13	1.4 (1.5)
Age of children	13	23.71 (15.2)
Working		
Full-time	8	35%
Part-time	2	9%
No	13	56%
Employment hours per week	10	35.7 (16.4)

**Table 2:** Clinical data of the participants in the questionnaire study (*N* = 23).

Parameters related to Spinal Cord Injury (SCI)	N	% of Sample
Level of SCI		
C2	1	4%
C3	4	17%
C4	10	43%
C5	8	34%
Cause of SCI		
Traffic accident	11	47%
Fall	4	17%
Sports-related	2	8%
Other	4	17%
Non-traumatic	2	8%
Wheelchair type		
Motorized	20	86%
Regular	3	13%
Main financial support		
Social security	12	52%
Ministry of Defense	10	43%
Work	1	4%
Years since SCI	23	20.6 (14.6)
Hours in bed per day (including sleeping)	23	10.9 (3.8)

#### Construction of questionnaires

We used parts of previously used questionnaires [7, 31–34] and added additional questions to create new questionnaires, tailored to the study design and to the special needs involved in interviewing patients with SCI.

The full questionnaire is provided in *Supporting Information S1 Text*. Briefly, it had three parts:

1. Items addressing demographic and clinical information
2. Items related to computer usage habits (e.g., daily duration of use), environments (e.g., home, office, etc.), and difficulties. To evaluate the most common purposes of computer use and other practical and emotional concerns, we used concurrent parts of the Matching Person and Technology questionnaire [33]. If the participants reported using an ATCC (e.g., a head-tracker [15]), they were asked to elaborate about their experience by answering the Quebec User Evaluation of Satisfaction with assistive Technology (QUEST) 2.0 questionnaire, which is widely used to assess assistive technology related satisfaction and is considered highly reliable and valid [35].
3. Open-ended questions targeting information on computer related needs, missing functions, and demands for improvement that participants raised with regard to the systems and/or assistive devices they were already using.

#### Procedure

Interviews took place either at the hospital or at the homes of participants. The interviewer read the structured questionnaires and recorded participant responses. For open-ended questions, the interviewer recapped the participant responses in short, clear sentences. The questionnaires were available in Hebrew, Greek, and English (the latter is provided in *Supporting Information S1 Text*). All the participants with SCI were interviewed using the Hebrew form.

#### Questionnaire results analysis

To analyze QUEST 2.0 results, average scores (which are in the form of a Likert scale from 1 to 5) were computed for the total questionnaire and for the ‘Device’ and ‘Services’ subscales. To assess the relative importance of different aspects of ATCCs, we used the results of the final part of the QUEST 2.0 questionnaire and an additional mini-questionnaire designed for this purpose (see *Supporting Information S1 Text*). For questionnaires using Likert scale-based responses, means and standard deviations (SD) were calculated. For descriptive questions (e.g., “Which operating system do you work with?”) and yes/no questions, frequencies were used. Clinically related requirements were then qualitatively derived from the final set of results.

In the Results sections, apart from demographic and clinical data, only the data that were used to draft clinically related requirements is reported. The rest of the data appears in *Supporting Information S2 Text*.

### Clinically related requirement assessment method

The clinically related requirements derived from the three sources were crossed and integrated. When requirements overlapped partially, new requirements were drafted, which included the reason for overlap. The requirements were then arranged into a list, categorized in accordance with the following ATCC-relevant dimensions: performance–basic operational aspects of the ATCC designed to achieve its purpose; personalization/adaptation–adaptability of the ATCC to personal characteristics of the user; interoperability–ability to use the ATCC to operate different platforms or devices; usability–extent to which devices were easy to learn and use; physiology–relevance of physiological characteristics to ATCC performance.

## Results

### Literature survey–Narrative review

Using the search words described in the Methods, 16 relevant articles were identified [6, 7, 36–49]. Since no article explicitly reported on clinically related requirements, these were extracted by contextual inference. For example, Caltenco et al. [7] reported that most of the people with SCI use a regular desktop or laptop computer running Windows Operating System (OS). In accordance, the following requirement was defined: "*Should be mainly targeted to work with a regular desktop or laptop computer running windows OS*.". The clinically related requirements derived from the narrative review are presented in Tables 3 and 4.

**Table 3:** Summary of general ATCC requirements derived from focus group, questionnaires, and narrative review.

**Focus group**	Enable capture of photos/videos, transfer to computer and uploading to website or application	Enable performance of several actions simultaneously	Include a personal identification system that does not require external assistance, such as biometric means of identification (retina, voice, facial features, etc.)	Enable work with new software or entrance to a website the user has not visited before, without requiring adaptations	Enable fast and accurate performance, that either makes up for or bypasses the need to use group muscles
**Questionnaires**	Reduce pain and fatigue in the neck and shoulders and increase overall effectiveness	Improve communication, work, and productivity	Enable operation of browsers, email clients, and word processors	Improve interpersonal interactions and relationships, attainment of education, and employment status/potential	Overcome keyboard typing related difficulties such as pressing two keys at the same time
	Operated by experienced computer operating audiences				
**Narrative review**	Enable interaction with other common operation systems or control of alternative electronic devices (e.g., TV remote, powered wheel chair)	Enable adaptation for people who demonstrate general cognitive problems such as attention and concentration deficits or compatibility with different ages and cognitive profiles			
**Focus group + narrative review**	Enable computer use in a variety of different body positions as well as easy change of position	Learn characteristics of the individual user			
**Narrative review + questionnaires**	Mainly targeted to work with a regular desktop or laptop computer running Windows OS	Comfortable, easy to use, durable, effective, non- invasive, productive; enable independent operation			
**Questionnaires** **+ focus group**	Designed to be operated while the computer is on a desk, and while the user is sitting in a regular or motorized wheelchair	Compatible with a wide range of computers (including tablets and smartphones) and softwares			

Blue–Performance, purple–Interoperability, Green—Personalization/Adaption, Red—Usability

**Table 4:** Summary of BCI/Gaze ATCC requirements derived from a focus group and a narrative review.

**Focus group**	"Translate" thought about an action into its required stages	Recognize the letters required to put together a word that the user is thinking of	Enable user to perform two actions simultaneously, such as watching content and responding to it	Distinguish between responses (eye gaze or brain signal) that are “noise” and those intended to relate intentional commands	Look as “normal” as possible
	Distinguish between movements that are “background noise” and those intended to relate intentional commands				
**Narrative review**	Use dry or at least “one drop” gel-less electrodes and a “push-button” user interface without the need for technical experts to setup and calibrate them manually	Demand a low number of calibration points	Include an EEG error-correction mechanism or algorithm	Provide a solution for users with potentially different inherent EEG characteristics, and accommodate variance in EEG characteristics due to mood, medication, and pain	

Blue–Performance, Red–Usability, Orange—Physiology

### Focus group

Due to length of the transcript, the following section includes contextual themes that were identified during the focus group discussion. The entire transcript used to extract clinically-related requirements can be found in *Supporting Information S3 Text*.

The contextual themes extracted are presented below, with examples of the statements that supported them:

#### Loss of independence of people with high tetraplegia

Persons with high SCI experience extreme loss of independence in almost all aspects of life: *“The extent of loss for the families is huge*. *Loss on top of loss*. *Patients speak of the physical ability of walking*, *but also about the loss of ability to control their sphincters*, *the loss of sexual function*, *even not being able to scratch their own nose*. *The losses are incomprehensible; I have tetraplegia patients that have wanted to kill themselves*.*”*

#### Significance of computer use for persons with SCI

In particular, the following topics were emphasized: (1) After returning home from their daily dealings, the computer enables SCI patients to regain control and independently manage a part of their life; (2) The computer becomes an anchor for functional independence in these individuals; (3) The computer is important as a tool for maintaining communication and social interaction for SCI patients. The computer is a central tool in getting SCI patients back into social participation during the phase of mental acclimation to their disabilities.

#### Computer use habits

(1) No particular type of computer platform (e.g., laptop versus desktop) or brand stood out as preferable to participants with SCI; (2) Sitting was the preferred body position for computer use: *"Most computer use is done sitting down*, *when you lie down it limits the movement; most functions are performed whilst sitting in a wheelchair*.*"*

#### Difficulties and barriers faced by people with SCI when operating computers

The group acknowledged that operation of computers was a challenge in itself: *“Everything that seems trivial to us*, *for them is a huge effort; if I think of a person that works with a head curser and has to lean back and move his head*, *and these are the only muscles that work*, *this is quite a big effort for him*. *He might be limited in terms of range of movement; these movements might cause him pain*.*”*

#### Specialized computer-use needs of people with SCI

(1) Securing privacy: *“I have a patient who*, *when she wants to check the status of her bank account*, *her caregiver is automatically exposed to her code*. *It’s not just the caregiver; there is something very public about it*.*”* (2) Identification of gentle muscle movements: *“(for computer use)…there is a need for accuracy using the muscles*, *the gentle movements are much more vulnerable than the gross muscles*, *and they need to make delicate movements to operate the computer*.*”*

Based on these themes and on additional discussion points, we identified, for example, that a proposed assistive device to be operated by gaze and brain waves should “*be able to ‘translate’ thought about an action into its required stages*.” Additional requirements are presented in Tables 3 and 4.

### Questionnaire-based cross-sectional study

#### Clinical and demographic information

The questionnaire study was completed by 23 participants with tetraplegia (16 males, 69%). Differences in gender proportions indicate that the sample is representative of the general SCI population, as 80% of people who have survived an SCI are men [50]. The mean age (and SD) was 49.22±15.8 years (range: 22–74). Additional details about employment and marital status, number of children, and employment status are provided in Table 1. Eighteen (77%) of the participants with tetraplegia had C4 and C5 spinal injuries, while the rest had higher injuries. The most common cause of injury was trauma (91.3%), of which the most common was traffic accidents (*n* = 11, 47%), followed by falls (*n* = 4, 17%).

Since ATCC can be used in bed, we asked the participant to report about the number of hours spent in bed per day. Since people with SCI report sleeping seven hours per day [51], we can deduct that people with SCI spend almost four extra hours not sleeping, in bed. Thus, although the questionnaires indicated that the preferred operating position is while sitting in a wheelchair, we did acknowledge that the assistive device should be designed to be operated in different positions. Interestingly, this requirement was extracted from the focus group and from the literature (See Table 1). Additional clinical data is provided in Table 2.

#### Social life and computer-use habits

The mini-questionnaire results revealed that most of the SCI participants reported a decline in their social life (60%), engagement in leisure activities (78%), and having regular mobility (73%). The platforms most owned by the participants were personal smartphones (69%) and laptop computers (65%). However, the platforms most used were desktop PCs (34.8%) and smartphones (30.4%).

The following results are from participants who reported owing a desktop or a laptop PC (18 participants, 78.3%). These participants reported using the computer for a mean of 5±3.4 hours daily (min 1, max 12) and having 20.5±9.1 years of computer use experience (min 10, max 40). The most commonly noted uses were ‘communication’ (82%) and ‘work and productivity’ (82%). The most popular computer applications were ‘internet browser’ (72%) and ‘word processor’ (72%), but when asked to indicate the most important one, all participants indicated the ‘Internet browser’ (100%). In addition, the vast majority of participants reported using Windows OS (94%) as their operating system. Participants also indicated that they used the computer mostly at home (88%), while sitting on a motorized wheelchair (61%). The most common aspects of life to which computers were reported to contribute were education (72%), interpersonal interactions and relationships (54%), and work and employment status/potential (54%). The most commonly reported locations of pain and fatigue caused by current operation method were the neck (55%) and the shoulders (38%).

When asked to indicate the most common difficulties they encountered while using computers, participants most often noted the following: typing on the keyboard (70%), pressing two buttons at once (60%), and zooming and panning (50%).

Results of the mini-questionnaires taken from the QUEST 2.0 revealed that the most commonly noted aspects of the ATCCs were comfort (77.8%), durability (66.7%), and effectiveness (66.7%). Results of another mini-questionnaire showed that the most important technical aspects were independent operation (85%), ease of use (71%), noninvasiveness (57%), and productivity (57%).

The clinically related requirements extracted from the questionnaires study are presented in Tables 3 and 4.

### Clinically related requirement assessment

The final list of requirements was divided into two tables. Table 3 presents general clinically related requirements that may be relevant to any ATCC. Table 4 presents specific requirements that are relevant to the objectives of the MAMEM project (i.e., requirements of ATCCs that are based on EEG reading or gaze behavior analysis.) Requirements that were identified using more than one method appear only in Table 3, as there were no repeated requirements relevant to the MAMEM project.

## Discussion

### Summary of findings

This paper describes a method that was used to collect data relevant to clinically related requirements of assistive devices from three sources: questionnaires administrated to potential users with SCI, a focus group of experienced clinicians and health professionals, and a review of the relevant empirical literature. The data were later used to extract the requirements of a novel ATCC, which can be considered the outcome measures. The results of the extraction processes (presented in Tables 3 and 4) constitute 29 requirements that were crossed between sources, rephrased if necessary, and arranged into categories. This made it possible to assess the relationships between the sources used, as well as their strengths and weaknesses and the advantages of using the combined method. For example, the results suggest that enabling computer use in different body positions is an important requirement of new ATCCs for persons with SCI, as it was independently extracted from two different methods.

### Relation to existing literature

#### Comparison to previously extracted clinically related requirements of new assistive devices

Papers defining clinically related requirements for the design of new assistive devices are sparse. Medola et al. [1] and Caltenco et al. [7] administrated questionnaires to individuals with SCI to assess their experiences and problems and reported results similar to ours, but without spelling out specific requirements.

Harmo et al. [24] used a similar, two-way method that included questionnaires, interviews, and a literature review to assess the needs and problems of elderly and disabled people in relation to home automation and service robots. However, they did not specifically indicate, or discuss, which conclusions were based on which method. In accordance with our results, communication and productively needs and physiological problems such as reduced fine motor skills also emerged as key factors in their analyses.

Lenker et al. [52] reviewed papers on consumer-identified assistive technology related outcome variables and conducted a number of focus groups with end users to assess these outcomes. Unlike the distinct requirement definitions presented in the current study, their outcomes were less specific, probably due to their inclusion of diverse end user cohorts (e.g., visual impairments and mobility impairments) and their reference to non-specific technologies.

Finally, Bright & Coventry [23] derived mobility related automated assistive technology requirements for older adults from interviews of care-givers and technology ‘tea parties’ (qualitative measures). Their analyses yielded socio-emotional and psychological design guidelines for future assistive devices. As in the present study, their conclusions were specific to a certain cohort. Interestingly, the qualitative methods in both our study and theirs revealed the requirement that the device should "look as normal as possible."

### Characterization of the clinically related requirements –The added value of each method

By reviewing the requirements derived from the three different methods, it is possible to identify distinctive attributes that each method produced. Requirements derived from the literature straightforwardly address specific clinical, physiological, and psychological attributes of SCI patients, as they are basically concise statements summarizing the results of the issues that have been addressed in the reviewed papers. The requirements derived from the focus group analysis mainly target the physical and emotional well-being of the patients. These requirements are less specific, constituting a set of general demands explicitly defined by the group members and not likely to reflect a technological perspective. Finally, the questionnaire driven requirements discuss daily computer use habits, difficulties, and characteristics and, as such, are specific and detailed.

#### Evaluation of the requirement extraction methods

Very few papers discuss the advantages of the different methods used to produce user requirements for assistive or medical devices, and no papers were found that discuss their disadvantages. Garmer et al. [53], for example, identified the strengths of usability tests and focus groups, but did not mention any of their weaknesses. We argue that an effective discussion of the advantages of the combined method must also address the disadvantages of the different methods. Thus, we describe the advantages and limitations of each extraction method in general, and specifically with respect to the present study.

In general, the literature review provided us with existing knowledge and with an idea of the general framework of prior efforts similar to ours. It has the advantage of being based on the most up-to-date published knowledge from clinical trials, systematic reviews, and meta-analyses, among other studies. Conversely, it is limited to published topics, it is likely that standard publication cycle times prevent the most recent technologies from appearing. Furthermore, the narrative review is not as comprehensive as a systematic review.

The focus group method produces firsthand opinions of experienced health professionals. Its advantages are the provision of comprehensive clinical perspectives based on the professional knowledge and experience of personnel who work closely with patients with SCI. As such, it can provide insights concerning end users that would not be acquired otherwise. For example, in the current study, the focus group raised the possibility that computer use could be a coping strategy for users with severe depression. One limitation of the focus group method is its dependence on the background of participants. In our study, the psychologist, social worker, and to some extent the nurse, referred mostly to emotional, social, and motivational aspects, while for other medical professionals, physical aspects of SCI were the baseline for discussion. Furthermore, the requirements extracted from this method may be theoretical and general. For example, in the present study, the moderator explicitly asked group members to try to suggest *every solution for SCI that they can imagine*, without limiting themselves. In her experience, limiting the group in advance was not recommended and should be done only during the discussions. Nonetheless, some of the stated requirements stemmed from "wishful thinking" that could greatly improve SCI computer use but might not be feasible for an ATCC. With respect to the current study specifically, the single meeting and small number of pre-selected members might have limited the scope of extracted requirements. We would also like to acknowledge the potential added value of focus groups of potential end users (e.g., Lenker et al. [52]), which were not included in the present study.

Questionnaires enable a direct, structured investigation with potential end users. Because information is extracted from the actual targeted end users, this method has the advantage of providing an authentic picture of their current use habits, difficulties, and needs. This is especially important in the field of computer use, in which habits, difficulties, and needs can change relatively fast and findings can become irrelevant fairly quickly. On the other hand, questionnaire-driven requirements can represent somewhat "narrow" thinking, since the questions themselves imposes limits on potential responses. This limitation is alleviated to some extent by the inclusion of open-ended questions, though responses to these questions lack standardization. Beyond this, the generalizability of the questionnaires used in the present study was limited, since they were not tested for reliability and validity. The convenience sampling method also limited the representativeness of outcomes. Finally, when considering data from SCI participant questionnaires, requirements should be derived in relation to upper limb deficits. For example, the needs of a patient with residual hand movements may differ from those of a completely immobile individual. However, due to the relatively small number of participants with C2 and C3 injury level (Table 2), we chose to generalize the conclusions to upper limbs deficits.

### The integrated three-way method

Several articles have discussed the use of more than one method to define requirements for new assistive technologies. Garmer et al. [54], for instance, emphasized the need for triangulation and an iteration process in designing medical devices, using both usability tests of existing equipment and human factor approaches (e.g., semi-structured interviews and cognitive walkthroughs). Garmer et al. [53] used a combination of usability tests and focus groups for identifying important usability aspects in the development process of medical equipment. They reported a small overlap between aspects identified using each of the methods, supporting our current findings. Shah et al. [55] proposed a theoretical framework for involving end users and professionals in the development of medical device technologies in different stages of the development lifecycle. Finally, Martin et al. [56] argued that despite financial and time constraints, different methods are effective in identifying different types of data, so more than one method should be used to assess user requirements.

The main advantage of the current method over previous ones (e.g. [1, 7, 23, 24, 52]) is that it incorporates three, rather than one or two, different methods for extracting clinically related requirements. The methods complement one another, such that each can overcome limitations of the others (e.g., narrative review outcomes can be somewhat outdated while questionnaire outcomes are as up-to-date as possible; focus group outcomes might lack external validity, which can be obtained using the other methods), maximizing the collection of relevant information.

In some cases, the different methodologies produced similar requirements [see Table 3]. It is proposed that such outcomes can be used as a measure of the quality of the methods employed. While repetitiveness is not necessarily an indicator of a requirement’s importance, it could potentially facilitate prioritization of needs. Furthermore, a considerable number of requirements appeared only once [Table 3], suggesting that each of the sources indeed had its own unique added value.

Our three-way method does not cancel out the limitations of each method, but it does provide a synergic approach to minimizing their impact. Naturally, it requires greater time and effort. Furthermore, the requirement assessment process cannot stand alone and should be followed up by complementary steps, such as usability studies [53], when device prototypes become available.

Clearly, we do not expect that all, or even most of the requirements suggested here will be met. However, by using the method presented here, it is now possible to organize, discuss, and prioritize the requirements, and to craft a work program accordingly when planning actual devices. We argue that in this manner, we can increase the level of certainty that the most effective and satisfactory assistive device is designed, in terms of the clinical needs of potential users with disabilities. In conclusion, we propose that similar approaches should be adopted in the future prior to designing, producing, and testing new ATCCs.

## Supporting information

S1 Text Depiction of the full questionnaire. (DOCX)

S2 Text Additional results from the questionnaire study. (DOCX)

S3 Text Focus group transcript. (DOCX)
