# Supplementary material for: Identification of clinically related requirements of a novel assistive device for people with a high spinal cord injury
Source: PLoS One. 2019 Jun 28;14(6):e0218393. doi: 10.1371/journal.pone.0218393 (PMC6599142; doi:10.1371/journal.pone.0218393)
Supplement: S1 Text — (DOCX) [file pone.0218393.s001.docx]

**Supporting Information S1 Depiction of the full questionnaire**

**Article title:** Identification of clinically related requirements of a novel assistive device for people with a high spinal cord injury

**Authors:** Amihai Gottlieb, Meir Plotnik, Racheli Kizony, Zoe Katsarou, Sevasti Bostantjopoulou and Gabi Zeilig

# S1 Text: Depiction of the full questionnaire

Rational for questionnaire:

The purpose of this questionnaire is to assess the computer use habits, difficulties and needs of subjects with spinal cord injury (SCI) under the demand of the first milestones of the MAMEM project.

Another issue regarding the questionnaires is the need for open and closed questions. The closed questions are designed to collect data appropriate for quantification and statistical analysis and the open questions are designed not to limit the subjects with their answers.

Since an appropriate questionnaire design to answer the specific study questions does not exist in the literature, some parts of the questionnaire were obtained from existing questionnaires^[[1]](#footnote-1)^ and some were created specifically for the MAMEM project objectives.

Questionnaire structure:

The questionnaire is based on three parts. First is the demographic & clinical information section. The second is designed to assess the computer related habits, environment and difficulties. The last part consists of open questions targeted to collect data regarding the needs, missing functions and demands of improvements that the subjects have from the current system and/or assistive device they are using.

Instructions for the interview:

The questionnaire should be filled by a research assistant interviewing the subject. All questions should be answered according to the order of their appearance. Some questions have specific instructions for the interviewer. These are italicized and appear in parenthesis below the question. When answering an open question, the interviewer should try to recap the subjects' remarks and summarize them in a few words.

IMORTANT – an interview can start only after an informed consent form has been signed by the interviewee.

Chapter I

Demographic and clinical information

a. Demographic information

Subject code: ____________________

(*Instructions to interviewer: create the code like this: SCI -#- XX.*

*# - according to participation order, XX – according to the first letters of the subject's first and last name. Make sure you match the subject's code to his/hers real name in a separate coding form. Subject's real name will be kept in the coded list together with the informed consent by the PI of each site according to privacy regulations)*

| Date:  ________________ | Age:  _________________ | Gender:  Male \ female |
| --- | --- | --- |
| Single \ married \  Divorced \ widower | Number of children:  _________________ | Ages of children:  ___________________  ___________________ |
| Educational years:  ____________ | Occupation:  _________________ | If employed:  Full time \ partial |
| Hours employed per week:  ________ |  |  |

b. Clinical information

1. Diagnosis: (Neurological level of injury (NLI) & American Spinal Cord Injury association – (ASIA) impairment scale (AIS) (International Standards for Neurological Classification of Spinal Cord Injury (ISNCSCI).

*(Instructions to interviewer: consult with MD/medical records)*

___________________________________________________________________

1. Reason of SCI:

Traumatic:

- Sport
- Assault
- Transport
- Fall
- Other:______________________________________________________

Non-traumatic:

- ____________________________________________________________

1. Years with SCI: ______________________________________________________
2. Are you in a motorized wheelchair? Yes / No
3. How many hours per day (approximately) do you spend in bed?
4. For how long have you been in a rehabilitation ward / day care ward, if any?

______________________________________________________________

1. Please specify the financial support (e.g. medical insurance) you are provided with, in order to address your disease).

*(Instructions to interviewer: only name the major sources of income)*

______________________________________________________________

1. In which of the following parts of the body do you present partial or complete immobility/numbness?

|  | Tongue | Jaw | Neck | Shoulders | Arms | Elbows | Wrists | Fingers |
| --- | --- | --- | --- | --- | --- | --- | --- | --- |
| Complete |  |  |  |  |  |  |  |  |
| Incomplete |  |  |  |  |  |  |  |  |

1. If you own and use a PC, can we take pictures or short videos **of** your computer workspace while you are using it**?** (This will be shared only with the research team**. In case the pictures or videos will be shared beyond the research team, your face will be blurred out.)**

Yes / No

Chapter II

Computer habits, working environment and difficulties

1. Computer use habits
2. How is your social life affected by your disability?

- My social life is normal.
- There is no significant effect on my social life apart from limiting energetic aspects, such as dancing.
- My social life is restricted and I do not go out as often.
- My social life is restricted to my home.
- I have no social life and feel lonely.

1. Have you any kind of hobby or recreational activity? Yes /No
2. If yes, please specify: _________________________________
3. How is your mobility outdoors affected by your disability?

- I travel frequently for needs / pleasure.
- I travel sometimes.
- I travel very rarely and only when there is an absolute need.
- I cannot travel and must stay home.

1. Of the following systems, which do you own?

- Desktop computer
- Laptop computer
- Tablet
- Smartphone

1. If you own more than one, which one do you use the most? _____________________________________________________
2. Do you use a PC? Yes / No

*(Instructions to interviewer: if the subject does not use a PC – even if he/she owns one - go straight to chapter III.)*

1. If so, how many hours (approximately) a day do you use it? ______________________________________________________
2. How many years of experience do you have using a computer?

______________________________________________________

1. Please indicate your main uses of your computer system and the three most important ones:

(*Instructions to interviewer: can choose more than one; mark an x next to the important three uses*)

| - Social participation (Facebook, forums, etc.) |  |
| --- | --- |
| - Productive activities (writing, editing, etc.) |  |
| - Study (on-line courses, articles, etc.) |  |
| - Games |  |
| - Recreation (movies, music, crossword puzzles, blogs, etc.) |  |
| - Communication (email, Skype, etc.) |  |
| - Activities of daily living (purchases, payments, bank, etc.) |  |
| - Information (Wikipedia, governmental sites, news, maps, etc.) |  |
| - Other: ____________________________________________________________________________________________________________________ |  |

1. Please indicate the main applications you use and the three most important ones:

*(Instructions to interviewer: can choose more than one; if chosen, name the main application the subject use; mark an x next to the important three)*

| - Internet browser: ___________________________________________ |  |
| --- | --- |
| - Email client:________________________________________________ |  |
| - Word processor:____________________________________________ |  |
| - Audio/video/image applications:_______________________________ |  |
| - Spreadsheets (e.g. excel ):____________________________________ |  |
| - Computer games:___________________________________________ |  |
| - Presentation software:_______________________________________ |  |
| - Programming/database:______________________________________ |  |
| - Media editing applications:____________________________________ |  |
| - Other: ____________________________________________________________________________________________________________________ |  |

1. Which operating systems do you work with?

- Microsoft Windows
- Unix / Linux
- Apple MacOS

1. How does computer use contribute to you in the following aspects?

| Please indicate the three most important aspects (*mark an x next to the aspect*s) | 1- not important at all, 5- very important) |  |
| --- | --- | --- |
|  | 5 4 3 2 1 | Interpersonal interactions and relationships |
|  | 5 4 3 2 1 | Close, intimate relationships |
|  | 5 4 3 2 1 | Educational attainment |
|  | 5 4 3 2 1 | Work and employment status/potential |
|  | 5 4 3 2 1 | Participation in desired community, social and civic activities |
|  | 5 4 3 2 1 | Autonomy and self-determination (making decisions) |
|  | 5 4 3 2 1 | Fitting in, belonging, feeling connected |
|  | 5 4 3 2 1 | Emotional well-being |
|  | 5 4 3 2 1 | Overall health |

1. Difficulties
2. Do you have difficulties performing the following on the computer system which you are using?

(*Instructions for interviewer: If a category is chosen, ask the subject to briefly specify what kind of difficulties*):

- Identifying the cursor on the screen _________________________________________________________________
- Moving the cursor on the screen _________________________________________________________________
- "Clicking" with the cursor _________________________________________________________________
- "Double clicking" with the cursor

_________________________________________________________________

- Selecting and dragging, resizing windows

_________________________________________________________________

- Zooming / Panning

_________________________________________________________________

- Using the keyboard _________________________________________________________________
- Identifying the letters on the keyboard _________________________________________________________________
- Typing with the keyboard _________________________________________________________________
- Using two keys at the same time

_________________________________________________________________

- Reading the words on the screen _________________________________________________________________
- Understanding how to use the assistive device software

_________________________________________________________________

- Opening a file on the computer

_________________________________________________________________

- Picking an item from a list or menu

_________________________________________________________________

- Navigating the directory structure _________________________________________________________________
- Perform a search on the computer or on the Web _________________________________________________________________
- Browsing/Navigating the internet

_________________________________________________________________

- Other:
  _________________________________________________________________

1. Fill up the following table:

| How difficult is it for you? (1- very difficult, 5- very easy) |  |  |
| --- | --- | --- |
| 5 4 3 2 1 | - Keyboard | How do you create a text on the computer and how easy it is? (*More than one option can be chosen*). |
| 5 4 3 2 1 | - By vocal dictating (a machine or a person) |  |
| 5 4 3 2 1 | - By touch |  |
| 5 4 3 2 1 | - Pointer and virtual keyboard |  |
| 5 4 3 2 1 | - Other : ______________________________ |  |
| 5 4 3 2 1 | - Mouse | How do you point on the screen and how easy it is? (*More than one option can be chosen*). |
| 5 4 3 2 1 | - Keyboard |  |
| 5 4 3 2 1 | - By touch |  |
| 5 4 3 2 1 | - Assistive device:   ______________________________ |  |
| 5 4 3 2 1 | - other _________________________ |  |

1. Description and evaluation of the current working environment:
2. Computer type:

- Stationary
- Portable

1. Computer location:

- On a desk
- Mounted on an arm
- Wheelchair tray
- Other: _________________________________________________________

1. Positioning while using the computer:

- Sitting on an armchair
- Sitting on special armchair
- Sitting on wheelchair
- Sitting on motorized wheelchair
- Standing
- Laying
- Other:_________________________________________________________

1. Common operating location/s:
   - Home
   - Work
   - Coffee shops
   - Other: __________________________________________________________
2. How does the current physical condition affect the following computer use aspects?

(*Instructions for interviewer: This question inspects the effect of the specific working environment in which the computer is operated, i.e. armchair/wheelchair/bed, desk, etc.)*:

| Not relevant | Completely | Substantially | Moderately | Mildly | No effect |  |
| --- | --- | --- | --- | --- | --- | --- |
|  | 5 | 4 | 3 | 2 | 1 | Comfort |
|  | 5 | 4 | 3 | 2 | 1 | Independence |
|  | 5 | 4 | 3 | 2 | 1 | Satisfaction |
|  | 5 | 4 | 3 | 2 | 1 | Pain |
|  | 5 | 4 | 3 | 2 | 1 | Speed of operation |
|  | 5 | 4 | 3 | 2 | 1 | Fatigue |
|  | 5 | 4 | 3 | 2 | 1 | Accuracy of operation |
|  | 5 | 4 | 3 | 2 | 1 | Endurance |
|  | 5 | 4 | 3 | 2 | 1 | Effectiveness |
|  | 5 | 4 | 3 | 2 | 1 | Ease of use |
|  | 5 | 4 | 3 | 2 | 1 | Enabling privacy |

1. Description and evaluation of assistive device/s
2. Do you use any assistive device for computer access (apart or instead from a standard keyboard and mouse)? Yes / No

(*Instructions for interviewer: if the subject does not use an assistive device, skip to chapter III*)

1. If so, what device/s?

(*Instructions for interviewer: can choose more than one, please specify brand*)

- Typing Stick:_______________________________________________________
- Mouthstick:________________________________________________________
- Chin joystick:_______________________________________________________
- Mouth joystick:_____________________________________________________
- Gaze tracker: ______________________________________________________
- Head tracker :______________________________________________________
- Speech recognition:_________________________________________________
- Mounting system (arms and support):___________________________________
- Other: __________________________________________________________________________________________________________________________________

1. How long have you been using this device/ these devices (*months/years*)? ____________________________________________________________________
2. Where was this assistive device fitted for you?

- During rehabilitation
- Vocational/assistive-device counseling center
- Private/commercial company
- Other:

____________________________________________________________________

1. Have you used a different assistive device in the past? Yes / No
2. If so, what kind of assistive device and why did you stop using it? ____________________________________________________________________

____________________________________________________________________

1. Please indicate which body parts do you use to operate the assistive device, and try to assess the pain and/or fatigue it causes after prolong use, if any:

(*Instructions for interviewer: can choose more than one*)

|  | Pain level after prolonged use (1 – no pain at all, 5 – extreme pain) | fatigue level after prolonged use (1 – no fatigue at all, 5 – extreme fatigue) |
| --- | --- | --- |
| - Tongue | 1 2 3 4 5 | 1 2 3 4 5 |
| - Eyes | 1 2 3 4 5 | 1 2 3 4 5 |
| - Jaw | 1 2 3 4 5 | 1 2 3 4 5 |
| - Neck | 1 2 3 4 5 | 1 2 3 4 5 |
| - Shoulders | 1 2 3 4 5 | 1 2 3 4 5 |
| - Arm | 1 2 3 4 5 | 1 2 3 4 5 |
| - Elbows | 1 2 3 4 5 | 1 2 3 4 5 |
| - Wrists | 1 2 3 4 5 | 1 2 3 4 5 |
| - Fingers | 1 2 3 4 5 | 1 2 3 4 5 |

*Instructions for interviewer:*

*Sections 7, 8, 9 and 10 are 'borrowed' from widely used questionnaires and their structure was kept.*

*Please fill out the following questionnaires regarding the assistive device the subject uses.*

*If the subject uses more than one assistive device, the following questionnaires refer to all of them as one 'system' which is actually the combination of all of them.*

*However, in case some questions in the questionnaires are answered in regards to a specific assistive device, please add in writing which device, next to the answer.*

1. Quebec User Evaluation of Satisfaction with assistive Technology

QUEST

(Version 2.0)

Technology device: _____________________________________

Date of assessment: ____________________________

The purpose of the **QUEST** questionnaire is to evaluate how satisfied you are with your assistive device and the related services you experienced. The questionnaire consists of 12 satisfaction items.

• For each of the 12 items, rate your satisfaction with your assistive device and the related services you experienced by using the following scale of 1 to 5.

• Please circle or mark the **one number** that best describes your degree of satisfaction with each of the 12 items.

• D**o not** leave any question unanswered.

• For any item that you were not "very satisfied", please comment in the section ***comments***.

Thank you for completing the QUEST questionnaire.

ASSISTIVE DEVICE

*How satisfied are you with,*

| 5 4 3 2 1 | 1. The **dimensions** (size, height, length, width) of your assistive device? |
| --- | --- |
| 5 4 3 2 1 | 2. The **weight** of your assistive device? |
| 5 4 3 2 1 | 3. The **ease in adjusting** (fixing, fastening) the parts of your assistive device? |
| 5 4 3 2 1 | 4. How **safe and secure** your assistive device is? |
| 5 4 3 2 1 | 5. The **durability** (endurance, resistance to wear) of your assistive device? |
| 5 4 3 2 1 | 6. How **easy** it is to use your assistive device? |
| 5 4 3 2 1 | 7. How **comfortable** your assistive device is? |
| 5 4 3 2 1 | 8. How **effective** your assistive device is (the degree to which your device meets your needs)? |

SERVICES

*How satisfied are you with,*

| 5 4 3 2 1 | 9. The **service delivery** program (procedures, length of time) in which you obtained your assistive device? |
| --- | --- |
| 5 4 3 2 1 | 10. The **repairs and servicing** (maintenance) provided for your assistive device? |
| 5 4 3 2 1 | 11. The quality of the **professional services** (information, attention) you received for using your assistive device? |
| 5 4 3 2 1 | 12. The **follow-up services** (continuing support services) received for your assistive device? |

• Below is the list of the same 12 satisfaction items. PLEASE **SELECT THE THREE ITEMS** that you consider to be **the most important to you**. Please put an X in the **3 boxes** of your choice.

- Dimensions
- Comfort
- Weight
- Effectiveness
- Adjustments
- Service delivery
- Safety
- Repairs/servicing
- Durability
- Professional service
- Easy to use
- Follow-up services

1. How important are the following attributes of the assistive device for you?

| Please indicate the three most important attributes (*mark an x next to the attribute*) | 1- not important at all  5 - very important |  |
| --- | --- | --- |
|  | 5 4 3 2 1 | noninvasiveness |
|  | 5 4 3 2 1 | setup time |
|  | 5 4 3 2 1 | independent operation |
|  | 5 4 3 2 1 | training time |
|  | 5 4 3 2 1 | cost |
|  | 5 4 3 2 1 | number of functions provided |
|  | 5 4 3 2 1 | response time |
|  | 5 4 3 2 1 | productivity |
|  | 5 4 3 2 1 | Ease of use |
|  | 5 4 3 2 1 | Aesthetics |
|  | 5 4 3 2 1 | Enabling privacy |

1. Assessment of Comfort

| 1- Extremely  uncomfortable  7- very  comfortable |  |
| --- | --- |
| 1 2 3 4 5 6 7 | Force required for actuation |
| 1 2 3 4 5 6 7 | Smoothness during operation |
| 1 2 3 4 5 6 7 | Effort required for operation |
| 1 2 3 4 5 6 7 | Accuracy |
| 1 2 3 4 5 6 7 | Operation speed |
| 1 2 3 4 5 6 7 | General comfort |
| 1 2 3 4 5 6 7 | Overall operation of input device |

Chapter III

Needs, missing functions and demands of improvements

1. Why don't you use a computer?

(*Instructions to interviewer: apply only to those who answered NO on question a.6 in chapter II*)

- I don't need to use a computer
- I don’t know how to use a computer
- I don't have a computer
- I cannot find a good assistive device
- It is too difficult in my condition
- I don’t like computers
- Other : ___________________________________________________________

1. If you could design your own assistive device for computer use or improve an existing one, what would it look like? What features would it have? If you chose to improve an existing one – how would you improve it?

Please detail:

_____________________________________________________________________

_____________________________________________________________________

_____________________________________________________________________

1. What operation of the computer you used to do prior to the disease, that you can't do now, you miss the most, if any?

*(Instructions to interviewer: ask this question only those who still use a computer or stopped using the computer due to disease. examples of operations: using the mouse, the keyboard, etc.)*

Please detail:

_____________________________________________________________________

_____________________________________________________________________

_____________________________________________________________________

1. What computer applications were you using prior to your disease that you now can't operate (or find very hard to operate) and miss the most, if any?

(*Instructions to interviewer: ask this question only those who still use a computer or stopped using the computer due to disease. if the subject hesitates, explain what is an application, e.g., Facebook, Word etc*.).

Please detail:

_____________________________________________________________________

_____________________________________________________________________

_____________________________________________________________________

1. Would you use an assistive device system based on mental commands? Yes/ No

(*Instructions to interviewer: if the subject hesitates, explain how such a system could function*).

Please detail:

_____________________________________________________________________

_____________________________________________________________________

1. What type of computer interaction do you think you could perform with an interface based on mental commands?

Please detail:

_____________________________________________________________________

_____________________________________________________________________

1. Would you use an assistive device system based on eye movements? Yes/ No

(*Instructions to interviewer: if the subject hesitates, explain how such a system could function*).

Please detail:

_____________________________________________________________________

_____________________________________________________________________

1. What type of computer interaction do you think you could perform with an interface based on eye-tracking?

Please detail:

_____________________________________________________________________

_____________________________________________________________________

1. Would you wear on your head an EEG recording device to facilitate controlling the computer with your thinking/mind? Yes/ No

(*Instructions to interviewer: if the subject hesitates, explain what a wearable EEG recorder looks like, e.g. how light it is*).

Please detail:

_____________________________________________________________________

_____________________________________________________________________

_____________________________________________________________________

1. Would you wear on your head special glasses designed to facilitate controlling the computer with your eyes? Yes/ No

(*Instructions to interviewer: if the subject hesitates, explain how these glasses would look and feel like*).

Please detail:

_____________________________________________________________________

_____________________________________________________________________

_____________________________________________________________________

1. Brooke, J. (1986). "SUS: a "quick and dirty" usability scale". In P. W. Jordan, B. Thomas, B. A. Weerdmeester, & A. L. McClelland. [Usability Evaluation in Industry](http://www.usabilitynet.org/trump/documents/Suschapt.doc). London: Taylor and Francis. -- *system usability scale.*

   Caltenco, H. A., Breidegard, B., Jönsson, B., & Andreasen Struijk, L. N. (2012). Understanding computer users with tetraplegia: Survey of assistive technology users. International Journal of Human-Computer Interaction, 28(4), 258-268.‏

   Demers, L., Ska, B., Giroux, F, & Weiss Lambrou, R. (1999). Stability and Reproducibility of the *Quebec User Evaluation of Satisfaction with assistive Technology* (QUEST). *Journal of ehabilitation Outcomes Measurement,* 3(4).

   International Organization for Standardization: ISO 9241-9 (2000). *Part 9: Requirements for non-keyboard input devices. Ergonomic requirements for office work with visual display terminals (VDT)*. Geneva, Switzerland: Author.

   Scherer MJ & Craddock G. *Matching Person & Technology* (MPT) assessment process. Technology & Disability, Special Issue: The Assessment of Assistive Technology Outcomes, Effects and Costs, 2002, 14(3), 125-131 [↑](#footnote-ref-1)
