# Supplementary material for: Identification of clinically related requirements of a novel assistive device for people with a high spinal cord injury
Source: PLoS One. 2019 Jun 28;14(6):e0218393. doi: 10.1371/journal.pone.0218393 (PMC6599142; doi:10.1371/journal.pone.0218393)
Supplement: S2 Text — (DOCX) [file pone.0218393.s002.docx]

**Supporting Information S2 Additional results from the questionnaire study**

**Article title:** Identification of clinically related requirements of a novel assistive device for people with a high spinal cord injury

**Authors:** Amihai Gottlieb, Meir Plotnik, Racheli Kizony, Zoe Katsarou, Sevasti Bostantjopoulou and Gabi Zeilig

# S2 Additional results from the questionnaire study

Of the 18 computer users, 13 participants reported using at least one ATCC (72.2%). The most used ATCCs were: typing sticks (70%) and mouth sticks (39%). These ATCCs are in use for a mean of 14.26 years (n=13 CI min 1 max 32). In addition, most of these ATCCs were fitted in the rehabilitation department during rehabilitation (54%) and the vast majority (92.3%) never tried a different ATCC since then. When asked which body parts they use for operating the ATCCs, the most common parts were the Neck (69.2%), the shoulders (53.8%) and the Jaw (53.8%). In regards with the computer interaction, the most common ways to move the cursor were using an ATCC (47%) followed by using a regular mouse (27%). To create text, most users indicated that they use a regular keyboard (50%) followed by using an ATCC (39%).

The final chapter of the questionnaire was intended to evaluate the reasons why participants do not own or use a computer, in case they mentioned this, and the attitudes towards a new gaze analysis and EEG reading based AD for computer operation.

For those who indicated that they do not own or use a computer (n=5, 21.7%), most of them answered they ‘find it too hard to operate a computer in their condition’ (80%) and that they ‘cannot find a good ATCC’ (60%). Regarding the participants attitudes towards a new ATCC based on EEG and eye-tracking, most of them answered they would use one which is based on mental commands (82.6%), yet when explained to them how this ATCC will work and what it will involve, only half of them (52%) said they would use it. When the participants were asked if they would use an ATCC based on gaze tracking, two thirds (65%) said they would use it, yet, when explained how this ATCC would work, more participants said they would use it (87%).
