# Supplementary material for: Identification of clinically related requirements of a novel assistive device for people with a high spinal cord injury
Source: PLoS One. 2019 Jun 28;14(6):e0218393. doi: 10.1371/journal.pone.0218393 (PMC6599142; doi:10.1371/journal.pone.0218393)
Supplement: S3 Text — (DOCX) [file pone.0218393.s003.docx]

**Supporting Information S1 Focus group transcript**

**Article title:** Identification of clinically related requirements of a novel assistive device for people with a high spinal cord injury

**Authors:** Amihai Gottlieb, Meir Plotnik, Racheli Kizony, Zoe Katsarou, Sevasti Bostantjopoulou and Gabi Zeilig

# S1 Text: Focus group transcript

## A. Background and methodological notes

As part of the development process of a computer use supporting system for SCI patients, one focus group was carried out amongst a variety of professional stakeholders.

The composition of professional participants:

- Doctor
- Nurse
- Social worker
- Health psychologist
- Occupational therapist
- Physiotherapist
- Speech therapist (participated in the first half our of the discussion only)

There were consistent differences in the themes raised by the different professionals. The psychologist and social worker, and to some extent the nurse, referred mostly to emotional, social and motivational aspects, whilst the other medical professionals used the physical aspects of SCI as their baseline for discussion.

Notes:

It should be taken into account that this analysis is based on one research unit only, with a very heterogenic nature. Hence, the findings should be viewed as indicative.

## B. Findings

1. Characteristics of SCI and the role of computer in rehabilitation

Reference to the type of injury:

The location of the injury determines its extent. Initially, professionals divide injuries into “4 limbs affected” vs "2 limbs affected”, with a further division according to the depth of the injury, namely, whether there is complete or incomplete loss of sensation. It appears that amongst those who experience incomplete loss of sensation, there is ongoing non-realistic hope that one day their condition will significantly improve. Paradoxically, this hope might hinder rehabilitation. There is danger that it might make these patients delay the search for supporting solutions or commence a rehabilitation process due to the hope that one day they will not need it.

*“The patients with complete loss of sensation deal with a very drastic and dramatic loss, which forces them to undergo a relatively brief grieving process during which they need to adjust to a different life. The less drastic patients are busy with getting rehabilitated; the experience of ‘the big change is just around the corner’ keeps them going”*

It appears that the incomplete loss of sensation population is very difficult to characterize, as the range of injuries and their level of severity is wide and highly diverse. Participants mentioned that SCI patients, especially those suffering from tetraplegia, suffer many difficult losses in so many areas of their lives. It appears that what they all have in common is an extremely difficult loss of independence. There is an indication that staff working with SCI patients experience frustration due to the gap between the severe consequences suffered by tetraplegia patients, and what actually can be done for them.

*“The amount of loss for the families is huge. Loss on top of loss. Patients speak of the physical ability of walking, but also about the loss of ability to control their sphincters, the loss of sexual function, even not being able to scratch their own nose. The losses are incomprehensible; I have tetraplegia patients that wanted to kill themselves”.*

*“It’s important to mention that there are very little functions to work on with these patients, even as a ‘therapist-patient’ team you are left very frustrated, you show up with high motivation and there is so little you can do”.*

The role of computer in the rehabilitation process is central and highly important, as described by one of the participants:

*“Because our life is one big computer, the meeting with the computer is the initial stage”*

During hospitalization, the computer enables injured individuals to make contact with those who were part of their pre-injury life: Family, friends, colleagues.

*“This is a stage in rehabilitation… if in the past we had a computer room, nowadays everybody’s got their own smartphone and tablet, availability is very high. Also, the initial role it plays in the rehabilitation process is important”*

After returning home the computer enables SCI patients to regain control and independently manage a part of their life. The computer becomes an anchor for functional independence for injured individuals. By doing so it touches upon a key difficulty of ‘loss of selfhood’ and hence provides a very significant rehabilitative tool. It is important to notice the central role of various mobile devices: Smartphone, tablet etc. as they are generally very common and enable SCI patients, computer dependent activity during hospitalization. The group has raised, unaided, the importance of the computer as a tool for maintaining communication and social interaction for SCI patients. The computer is a central tool in getting SCI patients back into social participation. The group reported that even for the patients themselves, social communication is probably the computer’s most central and important function.

Comment

Following this part of the discussion we focused on spinal cord patients injured in vertebra C4 upwards, both complete and incomplete.

2. SCI patients’ modes of computer use

Discussion revealed it difficult to characterize types of computers and systems at use with this population. SCI patients use a variety of computer types and it was impossible to point to any single type standing out. The type of supportive system for computer use the SCI patient will require mainly depends on his specific type of injury – which muscles he can move voluntarily, other health issues – respiration, vocal control and other variables such as the nature of desired activity, the level of knowledge and computer literacy pre-injury, age, type of computer used etc. Participants stressed that it is very difficult to give general responses regarding systems’ compatibility to SCI patients, rather it should be examined specifically per condition and per user.

*“Which system is preferable for C4… This is a question that needs to be referred to patients, I don’t know”*

There is an indication that patients who suffer from incomplete lack of sensation and can type, probably do not need a replacement system for a keyboard and mouse, yet they do experience a problem of lack of sensitivity.

There is an indication that the most common position for computer use is sitting down.

*“Most computer use is done sitting down, when you lie down it limits the movement, most function is done whilst sitting in a wheelchair. That’s in terms of positions but it really depends…”*

Supporting systems are characterized as tools aimed at providing a solution for the lack of ability to type and use a curser using the standard keyboard and mouse. Therefore, the types of different systems will in effect replace these functions. Participants mentioned:

- Gaze focus system (replaces both keyboard and mouse)
- Solutions for positioning the where there is controlled movement – an upside-down mouse, a head mouse, a hand or leg mouse, a mouse positioned on the chin, different types of joysticks.
- Systems based on giving orders through inhaling and exhaling
- Systems based on vocal control (most participants did not witness such systems in practice)

Participants look at the issue of computer use support systems from a broad perspective, taking into account everything it takes to achieve computer use experiences similar to those of healthy people. They state that in order to enable computer use in this population there is a need to adapt the work environment as well as providing accessibility for everything needed to use a computer. Namely, it’s not just about elements directly related with operating the computer, but with everything that enables a person to get into the position where they can operate a computer, starting with changing their body position – to sitting, proper holding of body parts (related to a sitting system), suitable motivation, the ability to turn a computer on and off, use additional accessories etc.

*“Sitting down a C4 is not simple; they need a sitting support system. If it’s a motorized wheelchair, the chair and back need to be able to lean back. Getting to a position of 90⁰ sitting down is not easy, to sit a C4 patient down steadily you need to tilt him back a bit”*

3. Characterizing difficulties with computer use

- Difficulty in achieving a suitable working position – even before they start to work, both the user and his/her carer need to exert effort into getting into the right working position.
- A need for adjusting the screen position – the position that enables body stability and movement control requires tilting the head slightly backwards and upwards, which requires adjusting the position of the screen.
- Low level of accuracy – because computer activation is based on gross movement such as that of neck or mouth muscles. With incomplete patients who have sensation in their fingers, movement is still less accurate compared to their pre-injury performance.
- Slow work – working on the computer is slower in comparison to that of a healthy person or pre-injury. Typing using a support system is typically very slow – letter by letter (for example using a virtual keyboard), compared to more fluent typing pre-injury. The use of the mouse curser is slower as well.
- Effort, pain and fatigue – due to the need to activate neck muscles and due to the constant need to refine and correct gross movement, computer use for SCI patients much focus and straining one’s eyesight.
- Limited computer work time– due to the effort required. It was estimated that most SCI patients can work up to 2 hours.
- Spasticity – might cause the hand to “lock” on instruments.
- Significant motivational difficulty – a high level of motivation is required, both to re-learn how to use the computer and also how to use the computer fluently, due to the effort, fatigue relative slowness of work.
- Complete lack of spontaneity – there is a need for preparation and planning both before and computer use.
- Need for constant assistance – for every action accompanying computer use, such as – getting a credit card out of the wallet to buy something online, taking a picture with your smartphone and transferring it to the computer, doing other basic actions such as turning the computer off and on – all this needs external assistance.
- Need for training for carers assisting the SCI patient with preparing and adjusting the work environment
- Funding difficulties

*"There is a need for accuracy using the muscles, the gentle movements are much more vulnerable than the gross muscles, and they need to make delicate movements for operating the computer”*

*“Everything that seems trivial to us, for them is a huge effort, if I think of a person that works with a head curser and has to lean back and move his head, and these are the only muscles at work this is quite a big effort for him. He might be limited in in terms of range of movement; these movements might cause him pain”*

*“The significant difficulty in typing is switching from fluent typing to letter-by-letter typing”*

*“You need to type letter after letter with your mouth, I keep imagining it… I have a patient that types with his eyes letter after letter, also virtuals [using a virtual keyboard] type letter-by-letter”*

*“A C4 patient and certainly a C3 patient have rather short sitting time- the amount time they can sit in front of the computer. You can’t leave them sitting for 4 hours. If you’ve arranged a sitting-down work environment it’s time-limited”*

4. Unsatisfied needs of SCI patients regarding computer use and ideas for improvements

- Lack of solutions for situations where there is a need to operate additional instruments accompanying computer use.

Today, vast parts of computer use are interactive, namely involving the creation and uploading of user content. These activities are especially relevant for social uses. Many social applications involve using videos / photos taken with equipment external to the computer. There is a need to take the photos, then transfer them to the computer and upload to the website or application. Most websites have a “share” or “like” button etc.

*“Someone to put credit card details for me, to open a photo in a dating website, I want to take a photo of myself, a flattering photo, social activity on the net should be more interactive”*

- Lack of ability to secure privacy

When using websites where privacy is of high importance and there is a need to type in passwords or credit card details, SCI patients must use an assistant. So, for example, to type in credit card details there is a need for someone to get the credit card out of the wallet and type in the details. Websites like National Insurance or Banks require password use. The lack of privacy hinders the SCI patient’s ability to use these sites freely and comfortably. The demand: A personal identification system that will not require external assistance – such as biometric means of identification (retina, voice, facial features etc.)

*“I have a patient that when she wants to check the status of her bank account, her carer is automatically exposed to her code. It’s not just the carer; there is something very public about it”*

- Specificity /lack of versatility of supportive solutions – today there is a need to make adaptations to different software and different types of computers

It appears that all existing solutions require a specific adaptation both to the type of computer and the different software. Hence, switching computers o starting to use a new software (e.g. for study purposes) requires making adaptations involving effort on the part of both user and his carer. The demand: Versatile systems that are compatible with a wide range of computers (including tablets and smartphones) and softwares, without needing to make a specific adaptation every time something changes in the nature of the user’s activities.

*“If you use a gaze focus system, you can’t just go to any computer and operate it, no, every adaptation requires someone else’s assistance, if you compare systems this should be one of the advantages”*

*“Say I took a selfie and I want to open it in Photoshop using a voice command. Can I do that?” “No”… “It appears to be possible only in the movies”*

*“I want to study fashion design, I want to use a graphic software”*

- Difficulty carrying out a number of actions simultaneously

It is difficult to carry out several actions simultaneously using a supporting system. For example, “dragging” and pressing a button simultaneously is difficult to accomplish

*“Everything that requires two hands [is difficult to do]”*

*“Dragging for instance…”*

*“[an additional participant] If I want to drag an object I use the shift key, but try to think how a person with tetraplegia would do that.”*

*“Press CTRL and highlight a few objects”*

*“Pressing CTRL+ALT+DLT”*

- Slowness of work and lack of accuracy

It appears that the problems of slowness of work and the difficulty to type precisely – choosing letters, or picking commands or buttons, do not currently have good enough solutions. The demand: Systems that will enable fast and accurate performance, that will either make up for or bypass the need to use gross muscles. In relation to this point most participants perceive a vocal-operated system as having a significant advantage

*“Vocal operation requires the least amount of effort” “It's the least cumbersome, seems less restrictive”*

Need to change body position with relative ease in order to prolong the limited work time the demand: Solutions that will enable using a computer in a variety of different body positions as well allowing for easy change of position.

- Difficulty doing joint computer work

The key example that came up was the lack of ability to play computer games and other joint activities with children or friends. In relation to that, speed is a limitation as well.

*“Joint activity, if someone has a kid and they want to play together they can’t”*

*“I want to play game consuls”*

*“If I want to play with someone or watch something with someone I can’. Someone needs to share the screen with me. I can’t play with someone”*

- Oversensitivity of supporting systems

Due to the relative coarse movement of SCI patients, the supporting systems are sometimes over sensitive, translating an unintentional movement into a command. Demands: A system that can distinguish between a movement that is “background noise” and a movement that is aimed at an intentional command.

5. Eye movement and brain wave control system: Comments, ideas and demands:

Participants were presented with a general outline of a supporting system for computer use based on gaze control and brain waves:

- Participants raised a concern that such a system might suffer from over sensitivity due to a difficulty in distinguishing between a brain wave aimed at carrying out a certain command on the computer and other unrelated thoughts / emotions that are manifested as brain waves. The sensitivity problem exists in current systems; however, it appears that the greatest concern here is that with this type of equipment there will be even greater difficulty in distinguishing between giving a command and “noise”, the reason being that content such as films, photos etc. involves much thought and emotional responses. And more generally, it is feared that the system will be sensitive to brain waves produced by the mental and emotional state of the user.

*“Is it influenced by an emotional state, like if I watch a film and get emotional or angry?”*

*“If I have a million thoughts in my head and the computer starts writing down the thoughts”*

*“Precision, be precise”*

*“The system needs to know how to filter what is meant for computer operation from emotional factors”*

- An additional concern relates to the need to break down a desired action into small multiple stages that need to be carried out in order to complete it. The need to break everything down into stages might make working on the computer laborious and strain full.
- Participants wished that the system be able to “translate” the thought of an action into its required stages. For example, the user will think about opening a new folder and the system will translate this thought to the required steps.

*“I’m thinking about the first, second and third steps, that can be confusing if I have a complex thought. If the system can break down that thought [into stages] that would be helpful”*

*“I think about the action and the system needs to break it down into processes, for example if I want to open a file I would think about it and the system will do it”*

The system that participants envision needs to bridge the gaps discussed earlier, therefore:

- Continuing on from the idea that the system will be able to break a thought about a computer operation into stages, participants wish the system could overcome the slow typing problem, by having the user think of a word and the system would know which letters are required to put it together.

*“If I want to write something ... I don’t want to have to break the word down in my head into letters but rather have a word completion function”*

- In order to avoid typing errors, participants had additional ideas for improvement: A system that familiarizes itself with the characteristics of the individual user, the option to choose between different templates according to the type of text the user wishes to create, having an editing option in case the system is not sure of the user’s intent, presenting spelling options for the user to choose from.

*“A system that will learn my style of writing”*

*“In order to improve the speed of work there is a need to establish templates, like WhatsApp that completes words for the user, or like I have a fixed shopping list or like Google which learns the way we use the internet. Just like Google learns our pattern of activity – that would make working on the computer easier and faster”*

- Participants wish the system would enable carrying out two actions simultaneously such as watching a content and responding to it

*“Like working with two windows open”*

*“I want to watch MasterChef and vote for my candidate”*

- Participants would like the system to be versatile and enable working with new software or visit a website the user has not visited before, without having to make adaptations. A system that is adapted to a variety of operating systems and types of computers.
- The system needs to be usable in both sitting and lying down positions.
- Participants wish the system to look as “normal” as possible

*“The closest to normal as possible, even in terms of operation, that I would have to do as little adaptation as possible, like in terms of what the curser looks like, and in terms of the different commands”*

*“It needs to look nice, be light-weight, suitable for sitting and lying down. Have a normative appearance, like the headphones young people wear these days”.*

The issue of lack of appropriate sensitivity and more generally the ability to enable better control of the system came up in discussion repeatedly.

- Some participants initially thought that the eye gaze system would be used to move the cursor (replacing the mouse), while the brain wave system would be used to give commands such as “open”. As the discussion unfolded, the group suggested that the question as to which system is used for which operation depends on the type of activity the user is carrying out at the time. The decision will depend on the conditions under which there is a better distinction between a response (of eye gaze or brain waves) that is “noise” and a response that is intended for carrying out a command on the computer, as well as which system would interfere less in carrying out the key operation. For example, in operations where eye gaze is the main function- like watching a video, it might be better that computer control would be carried out using brain waves, whereas with operations where there is much thought and emotional response it might be preferable that operations would be done via eye gaze.
- Based on participants’ comments it can be concluded that there is a need to establish which system would provide a better level of sensitivity and accuracy and prevent interference with the main activity-per use.

Additional comments:

Some of the issues raised in the discussion require further expansion and in-depth exploration through direct testing with users, for quantification and validation purposes Social legitimacy – one of the needs raised – especially by the health psychologist – is the use of computer, which minimizes as much as possible the SCI patient’s difference. For example, the possibility of sitting at a café with a laptop, without having to use a system that attracts too much attention or looks strange to people. The system should have a good appearance which creates a sense of normality.
